# *DAAM2* gene variants p.(Asp165Glu) and p.(Asn614ThrfsTer2) in pediatric cryptorchidism: a hypothesis-generating pilot case-control study from the Eastern Black Sea region of Türkiye

**DOI:** 10.55730/1300-0144.6340

**Published:** 2026-06-30

**Authors:** Onur YALÇIN, Çağrı DOĞAN

**Affiliations:** 1 Department of Pediatric Surgery, Faculty of Medicine, Ordu University, Ordu, Turkiye; 2 Department of Medical Genetics, Faculty of Medicine, Ordu University, Ordu, Turkiye

**Keywords:** Cryptorchidism, *DAAM2*, androgen receptor, testicular descent, formin, genetic association study

## Abstract

**Background/aim:**

Cryptorchidism is the most common congenital anomaly of the male genitourinary system, characterized by a multifactorial etiology. The disheveled-associated activator of morphogenesis 2 (*DAAM2*) gene regulates androgen receptor (AR)-dependent transcription; its variants are associated with partial androgen insensitivity. Since the inguinoscrotal phase of testicular descent is androgen-dependent, we hypothesized that *DAAM2* variants contribute to cryptorchidism susceptibility. This pilot study, designed to generate hypotheses rather than to establish association, investigated the frequency of two functionally characterized *DAAM2* variants, p.(Asp165Glu) and p.(Asn614ThrfsTer2), in pediatric cryptorchidism.

**Materials and methods:**

This prospective case-control study included 40 male pediatric cryptorchidism patients and 39 age- and sex-matched healthy male controls. Genomic DNA was extracted from peripheral blood. The p.(Asp165Glu) variant was genotyped by TaqMan allelic discrimination and p.(Asn614ThrfsTer2) by ARMS-PCR with melting curve analysis. Genotype distributions were compared using linear-by-linear association testing with exact p-values, and odds ratios (ORs) with 95% confidence intervals (CIs) were calculated by binary logistic regression.

**Results:**

Heterozygous p.N614Tfs*2 carriage was more frequent in cryptorchidism patients (12.5%, n = 5) than in controls (2.6%, n = 1), with an unadjusted OR of 5.43 (95% CI: 0.6048.78, p = 0.131). Heterozygous p.D165E frequency was similar in both groups (7.5% vs. 7.7%; OR: 1.03, 95% CI: 0.19–5.45, p = 0.974), whereas homozygous p.D165E was identified exclusively in 2 of 40 cryptorchidism patients (5.0%) and in none of the controls, resulting in complete separation in the regression model.

**Conclusion:**

Although statistical significance could not be reached due to the limited size of the study group, the observed fivefold increase in heterozygosity of p.(Asn614ThrfsTer2) and the presence of homozygosity of p.(Asp165Glu) in cryptorchidism cases contribute to the literature. These data suggest that *DAAM2* variants may play a potential role in the etiology of cryptorchidism.

## 1. Introduction

Cryptorchidism, defined as the failure of one or both testes to descend into the scrotum, is the most prevalent congenital anomaly of the male genitourinary system [1,2]. The condition affects approximately 3% of full-term and 15%–30% of preterm male newborns, with the prevalence decreasing to 1%–2% by the end of the first year of life due to spontaneous postnatal descent [2,3]. Persistent cryptorchidism is associated with increased risks of impaired spermatogenesis, testicular germ cell tumors, testicular torsion, and inguinal hernia in later life [4]. Familial aggregation studies have demonstrated substantial heritability, with first-degree male relatives of index cases showing a threefold to tenfold increase in recurrence risk, consistent with a strong genetic contribution [5].

Testicular descent is a biphasic process. The initial transabdominal phase is primarily regulated by insulin-like factor 3 (INSL3) secreted from fetal Leydig cells and its receptor RXFP2, which drives gubernacular outgrowth and cranial suspensory ligament regression [2,6]. The subsequent inguinoscrotal phase, which occurs between weeks 25 and 35 of gestation, is predominantly androgen-dependent and requires functional androgen receptor (AR) signaling for gubernacular shortening and migration of the testis through the inguinal canal into the scrotum [2,6]. Disruptions at any point along the hypothalamic–pituitary–gonadal axis or within the androgen signaling pathway can therefore result in incomplete testicular descent. Despite the recognized roles of INSL3, RXFP2, and AR, identifiable monogenic mutations in these genes account for only a small proportion of nonsyndromic cryptorchidism, indicating that additional genetic factors remain to be characterized [2,7].

The disheveled-associated activator of morphogenesis 2 (*DAAM2*) gene, located on human chromosome 6p21.2, encodes a member of the formin family of cytoskeletal regulators [8,9]. Formins are characterized by conserved formin homology (FH) domains FH1 and FH2, through which they nucleate and processively elongate linear, unbranched actin filaments [9]. *DAAM2* has been implicated in multiple developmental and cellular processes, most notably in canonical and noncanonical Wnt signaling and dorsal patterning of the developing spinal cord [8]. Biallelic loss-of-function variants in *DAAM2* cause autosomal recessive steroid-resistant nephrotic syndrome type 24 through impaired podocyte actin dynamics [9]. In addition, a maternally inherited 167-kb partial duplication encompassing *DAAM2* and *MOCS1* was previously reported in a patient with 46,XY disorder of sex development and complete gonadal dysgenesis, suggesting a role of *DAAM2* in gonadal and urogenital development [10].

A landmark study published in 2023 by Knerr et al. provided direct mechanistic evidence linking *DAAM2* to androgen signaling [11]. Using a combination of patient-derived variant screening and high-resolution microscopy, these researchers identified heterozygous functional *DAAM2* mutations in two unrelated 46,XY patients with partial androgen insensitivity syndrome presenting with hypospadias, micropenis, and normal serum testosterone [11]. The first patient carried a frameshift variant, p.(Asn614ThrfsTer2) (c.1840_1841insC), predicted to yield a truncated DAAM2 protein lacking the FH2 domain, while the second patient harbored a missense variant in the GTPase-binding domain, p.(Asp165Glu) (c.495C>G, rs746895842) [11]. Functional analyses demonstrated that *DAAM2* is enriched in the nucleus, colocalizes with the AR upon dihydrotestosterone (DHT) stimulation, and mediates the assembly of actin-dependent transcriptional condensates with the AR [11]. Both variants significantly reduced DHT-induced AR transcriptional activity, establishing DAAM2 as a coregulator of AR-mediated gene expression [11].

Given that AR signaling is essential for the inguinoscrotal phase of testicular descent, functional *DAAM2* variants impair AR-dependent transcription [11], and cryptorchidism and hypospadias share a common origin in disrupted fetal androgen action within the broader testicular dysgenesis syndrome framework [1], we hypothesized that *DAAM2* variants may also contribute to the pathogenesis of cryptorchidism. To our knowledge, no previous study has examined the association between *DAAM2* variants and cryptorchidism. The aim of the present study was to investigate the frequency of two functionally characterized *DAAM2* variants, p.(Asp165Glu) and p.(Asn614ThrfsTer2), in a cohort of pediatric patients with cryptorchidism compared to healthy controls.

## 2. Materials and methods

### 2.1. Study design and ethical approval

This study was designed as prospective research and conducted at a tertiary care training hospital between 11 October 2024 and 19 August 2025. Ethical approval was granted by the Ordu University Clinical Research Ethics Committee (protocol number: 2024/141; approval date: 11 October 2024). The study was performed in accordance with the Declaration of Helsinki. Written informed consent was obtained from all participants; for all pediatric participants, written informed consent was provided by a parent or legal guardian, and assent was obtained from older children as appropriate.

### 2.2. Study population

The cryptorchidism group consisted of 40 male pediatric patients with unilateral or bilateral undescended testes diagnosed by the Department of Pediatric Surgery and subsequently referred to the Department of Medical Genetics for genetic evaluation. Diagnoses were confirmed by clinical examination and, where indicated, scrotal/inguinal ultrasonography. Patients with known chromosomal anomalies, recognized syndromic features, or disorders of sex development were excluded from the study.

The control group comprised 39 age- and sex-matched healthy male children referred to the Department of Medical Genetics for familial Mediterranean fever (FMF) genetic testing (*MEFV* analysis). These children had been referred because of recurrent, self-limited febrile episodes and/or recurrent abdominal pain rather than because of any confirmed systemic disease; given the frequency of oligosymptomatic, incomplete, and atypical FMF presentations in our regional population, *MEFV* sequencing was initiated without first requiring fulfillment of the Tel Hashomer clinical criteria. The members of the control group had no personal or family history of cryptorchidism, hypospadias, or any other genitourinary anomaly. A confirmed clinical or molecular diagnosis of FMF was therefore not an exclusion criterion for control group enrollment. Apart from the recurrent febrile and/or abdominal symptoms that prompted *MEFV* testing, the members of the control group had no known chronic systemic, endocrine, or developmental disorders and, importantly, no condition known to affect testicular descent or genital development. Given the referral criteria for the control group, this group was not entirely free of the recurrent autoinflammatory phenotype, but FMF does not play a role in the etiology of undescended testicles.

### 2.3. Blood sample collection and DNA extraction

Peripheral venous blood samples (2 mL) were collected from all participants into tubes containing ethylenediaminetetraacetic acid (EDTA). Genomic DNA was extracted using the DiaRex Whole Blood Genomic DNA Extraction Kit (catalog number BLD-5295; Diagen, Ankara, Türkiye), according to the manufacturer’s protocol. The extraction procedure included cell lysis, proteinase K digestion, ethanol precipitation, and silica column-based purification. DNA concentration and purity were assessed spectrophotometrically using a Colibri Microvolume Spectrometer (Titertek-Berthold, Pforzheim, Germany). DNA samples were stored at −20 °C until analysis.

### 2.4. Genotyping of *DAAM2* variants

Variants are reported relative to the MANE Select transcript *DAAM2* NM_001201427.2 (protein NP_001188356.1) on GRCh38, in accordance with current Human Genome Variation Society recommendations. p.(Asp165Glu) corresponds to NM_001201427.2:c.495C>G (NC_000006.12:g.39867576C>G; rs746895842) and p.(Asn614ThrfsTer2) to NM_001201427.2:c.1840_1841insC.

#### 2.4.1. Genotyping of p.(Asp165Glu) (c.495C>G, rs746895842)

The p.(Asp165Glu) missense variant was genotyped using a custom-designed TaqMan probe-based allelic discrimination assay. Primers and allele-specific minor groove binder (MGB) probes were designed as follows: forward primer 5′-CGTACTCAACCTATGCGTTT-3′; reverse primer 5′-AGCTAAAACATGAGCACGTC-3′; wild-type probe 5′-FAM-GGATCATGCTACTTGTG-MGB-3′; and mutant probe 5′-HEX-GGAACATGCTACTTGTG-MGB-3′. Polymerase chain reaction (PCR) cycling conditions consisted of an initial denaturation at 95 °C for 10 min, followed by 40 cycles of 95 °C for 10 s and 60 °C for 30 s. Allelic discrimination was performed by endpoint fluorescence analysis on a CFX-96 real-time PCR system (Bio-Rad Laboratories, Hercules, CA, USA).

#### 2.4.2. Genotyping of p.(Asn614ThrfsTer2) (c.1840_1841insC)

The p.(Asn614ThrfsTer2) frameshift variant was analyzed using amplification refractory mutation system (ARMS)-PCR combined with high-resolution melting curve analysis. The following primers were used: common forward primer 5′-GCGCGAGCTCCTCCTTGTTTAGGAATGGGA-3′; wild-type reverse primer5′-GATACGCTGCTTTAATGCCTTTAACCTTCTCATCCTTTAAAATCTT-3′; and mutant reverse primer 5′-CGGCCACCTTCTCATCCTTTAAAATCTA-3′. Cycling conditions consisted of initial denaturation at 95 °C for 10 min (1 cycle), followed by 40 cycles of 95 °C for 10 s and 54 °C for 30 s. Melting curve analysis was performed from 65 °C to 95 °C with increments of 0.5 °C. All reactions were carried out using TaqProbe 2X qPCR MasterMix (SensiFAST, Bioline, Memphis, TN, USA) on the same Bio-Rad CFX-96 real-time PCR system.

#### 2.4.3. Quality control

Various measures were taken to ensure genotyping accuracy. Each assay was optimized from start to finish before clinical samples were processed, including MasterMix composition, template input, primer/probe concentrations, and thermal cycling conditions, and only DNA samples that met spectrophotometric concentration and purity criteria were included in the analysis. Each plate contained a no-template control and positive controls with known genotypes, and for each sample, amplification curves, end-point allele discrimination clusters (TaqMan assay), and high-resolution melting profiles (ARMS-PCR assay) were examined separately. A result was classified as indeterminate if it showed overlapping or poorly resolved allele resolution clusters, an atypical melting profile, or an inconsistent amplification curve; such samples were reanalyzed from the original DNA and, if a clear genotype could still not be obtained, they were excluded from statistical analyses. To assess reproducibility, a randomly selected 10% of samples were regenotyped in independent PCR reactions, with 100% concordance between replicates. Under these conditions all 79 samples yielded unambiguous, concordant genotypes for both variants; the call rate was therefore 100% and the failed-genotyping rate 0%, and no sample required exclusion. The laboratory personnel who performed DNA extraction and genotyping were blinded to case/control status throughout the procedures; samples were processed and reported on a coded, per-sample basis, and group assignment was linked afterwards by the investigator for analysis. Genotypes were not independently confirmed by an orthogonal method such as Sanger sequencing.

### 2.5. Statistical analysis

Statistical analyses were performed using IBM SPSS Statistics 26.0 (IBM Corp., Armonk, NY, USA). Additional analyses, including Hardy–Weinberg equilibrium testing, Firth penalized logistic regression, and power analyses, were conducted with R software (R Foundation, Vienna, Austria). Categorical variables were summarized as frequencies (n) and percentages (%).

Genotype distributions of the *DAAM2* variants were compared between the cryptorchidism and control groups. The ordinal genotype trend was assessed using linear-by-linear association testing. Because expected cell counts were below 5 in several cross-tabulations, exact p-values were reported instead of asymptotic p-values. The Fisher exact test was used for 2 × 2 comparisons and the Fisher–Freeman–Halton test with Monte Carlo simulation was used for larger contingency tables.

Hardy–Weinberg equilibrium was assessed in the control group for each variant using an exact test. Observed genotype counts were compared with expected genotype counts calculated from allele frequencies in the control group. Minor allele frequencies, genotype frequencies, and variant genotype presence rates were also calculated. Variant genotype presence was defined as the presence of either a heterozygous or homozygous variant genotype, while the wild-type/homozygous reference genotype was used as the comparison category.

Allelic, dominant, and, where applicable, recessive genetic models were evaluated. In the dominant model, heterozygous and homozygous variant genotypes were combined and compared with the wild-type/homozygous reference genotype. The recessive model was evaluated only when the homozygous variant category was applicable. Between-group comparisons were performed using the Fisher exact test. Crude odds ratios (ORs) and 95% confidence intervals (CIs) for the association between genotypes and cryptorchidism risk were estimated using binary logistic regression. However, because of rare variant distribution, sparse cell counts, and complete separation in the homozygous p.(Asp165Glu) comparison due to the absence of individuals with the homozygous variant in the control group, Firth penalized logistic regression was additionally applied to obtain finite and less biased effect estimates. Exact logistic regression methods were used where appropriate. Cohort allele frequencies were also evaluated in relation to population reference data obtained from gnomAD v4 (Broad Institute, Cambridge, MA, USA).

A priori sample size determination was performed using G*Power software (Heinrich Heine University, Düsseldorf, Germany). Because no prior study had evaluated the association between *DAAM2* variants and cryptorchidism, the sample size calculation was not intended as a confirmatory, literature-derived association estimate but rather as a pragmatic estimate of the minimum detectable effect size within a hypothesis-generating pilot study design. Therefore, for chi-square testing of associations in a 2 × 2 table, a two-sided α level of 0.05, 80% power, and a medium-to-large effect size of w = 0.32 were assumed. Under these assumptions, the minimum total sample size was calculated as 78 participants.

Post hoc power analyses were additionally performed for the observed variant genotype differences. These analyses were based on the difference in variant genotype presence rates between the cryptorchidism and control groups and were conducted as two-proportion comparisons with a two-sided α level of 0.05. Effect size was calculated using Cohen’s h. The achieved statistical power with the current sample size was 0.430 for pN614pdfx2 and 0.110 for pD165E. The probability of type II error was calculated as 1 –power, yielding type II error probabilities of 0.570 for pN614pdfx2 and 0.890 for pD165E. The required sample size per group to detect the observed variant genotype differences with 80% and 90% power was also estimated (Table 1). A two-sided value of p < 0.05 was considered statistically significant for all analyses.

## 3. Results

A total of 79 participants were included in the analysis, comprising 40 pediatric patients with cryptorchidism and 39 healthy controls. The baseline clinical characteristics of the cryptorchidism cohort are summarized in Table 2.

Hardy–Weinberg equilibrium was first assessed in the control group for both *DAAM2* variants using an exact test. For p.(Asp165Glu), the observed genotype counts were 36 for the wild-type/homozygous reference genotype, 3 for the heterozygous genotype, and 0 for the homozygous variant genotype. The corresponding expected genotype counts were 36.06, 2.88, and 0.06, respectively. No significant deviation from Hardy–Weinberg equilibrium was detected for p.(Asp165Glu) (exact p = 1.000). For p.(Asn614ThrfsTer2), the observed genotype counts were 38 for the wild-type/homozygous reference genotype, 1 for the heterozygous genotype, and 0 for the homozygous variant genotype. The corresponding expected genotype counts were 38.01, 0.99, and 0.01, respectively. This variant also showed no significant deviation from Hardy–Weinberg equilibrium (exact p = 1.000) (Table 3).

The genotype distributions of the *DAAM2* p.(Asn614ThrfsTer2) and p.(Asp165Glu) variants according to study group, together with the linear-by-linear association results, are presented in Table 4. For the p.(Asn614ThrfsTer2) variant, the heterozygous genotype was more frequent in the cryptorchidism group than in the control group [5/40 (12.5%) vs. 1/39 (2.6%)]. The wild-type/homozygous reference genotype was observed in 35 of 40 patients with cryptorchidism (87.5%) and in 38 of 39 controls (97.4%). No homozygous variant genotype for p.(Asn614ThrfsTer2) was detected in either group. The linear-by-linear association test did not detect a statistically significant ordinal trend across genotype categories (χ^2^ LBL = 2.743, exact p = 0.201).

For the p.(Asp165Glu) variant, heterozygous genotype frequencies were similar between the two groups [3/39 (7.7%) in the control group and 3/40 (7.5%) in patients with cryptorchidism]. The homozygous variant genotype was detected exclusively in the cryptorchidism group, in 2 of 40 patients (5.0%), and it was absent in all 39 controls. The linear-by-linear association test did not identify a statistically significant linear trend across genotype categories (χ^2^ LBL = 1.163, exact p = 0.423).

The binary logistic regression estimates for the association between genotypes and cryptorchidism risk are presented in Table 5. In binary logistic regression, the heterozygous p.(Asn614ThrfsTer2) genotype was associated with approximately fivefold higher odds of cryptorchidism compared to the wild-type/homozygous reference genotype; however, this estimate did not reach statistical significance and had a wide CI crossing unity (OR: 5.43, 95% CI: 0.60–48.78; p = 0.131). The heterozygous p.(Asp165Glu) genotype was not associated with cryptorchidism risk (OR: 1.03, 95% CI: 0.19–5.45; p = 0.974). For the homozygous p.(Asp165Glu) category, complete separation occurred because no homozygous variant individuals were present in the control group; therefore, a reliable OR could not be estimated.

Because of sparse cell counts and zero cells in some genotype categories, genotype effects were additionally evaluated using the Fisher exact test, exact logistic regression, and Firth penalized logistic regression (Table 6). The heterozygous p.(Asn614ThrfsTer2) genotype was numerically more frequent in the cryptorchidism group, but statistical significance was not reached by any method. The OR was 5.33 (95% CI: 0.56–263.02; p = 0.201) in Fisher exact analysis, 4.53 (95% CI: 0.50–228.78; p = 0.225) in exact logistic regression, and 3.97 (95% CI: 0.74–40.17; p = 0.111) in Firth penalized logistic regression. Since no homozygous variant genotype was observed for p.(Asn614ThrfsTer2), an OR could not be calculated for this category.

No significant association was observed for the heterozygous p.(Asp165Glu) genotype. In Fisher exact analysis, the OR was 1.03 (95% CI: 0.13–8.21; p = 1.000); in exact logistic regression, the OR was 1.08 (95% CI: 0.12–10.25; p = 1.000); and in Firth penalized logistic regression, the OR was 1.03 (95% CI: 0.21–5.16; p = 0.972). The homozygous p.(Asp165Glu) variant genotype was observed only in the cryptorchidism group. For this comparison, the OR was infinite in Fisher exact analysis (95% CI: 0.18–∞; p = 0.493); in exact logistic regression, the OR was 2.31 (95% CI: 0.17–∞; p = 0.479); and in Firth penalized logistic regression, the OR was 5.12 (95% CI: 0.40–719.56; p = 0.228). The overall genotype distribution of p.(Asp165Glu) did not differ significantly between the groups (Fisher exact p = 0.595).

## 4. Discussion

In this case-control study, we examined the potential contribution of two functionally characterized *DAAM2* variants, p.(Asn614ThrfsTer2) and p.D165E, to the etiology of nonsyndromic pediatric cryptorchidism. To our knowledge, this is the first investigation to evaluate *DAAM2* variants in a cryptorchidism cohort. The most noteworthy observations were an approximately fivefold higher frequency of p.(Asn614ThrfsTer2) heterozygous carriage in patients compared to controls and the exclusive detection of p.(Asp165Glu) homozygosity in the cryptorchidism group.

We examined the potential contribution of two functionally characterized *DAAM2* variants, p.(Asn614ThrfsTer2) and p.D165E, to the etiology of nonsyndromic pediatric cryptorchidism. To our knowledge, this is the first investigation to evaluate *DAAM2* variants in a cryptorchidism cohort. The principal observations of a numerically higher frequency of p.(Asn614ThrfsTer2) heterozygous carriage in patients and the exclusive detection of p.(Asp165Glu) homozygosity in patients did not reach statistical significance and should be regarded as hypothesis-generating rather than as evidence of association.

### 4.1. Rationale for *DAAM2* as a candidate gene in cryptorchidism

The selection of *DAAM2* as a candidate gene for cryptorchidism was motivated by the recent demonstration that DAAM2 acts as a coregulator of AR-dependent gene expression. Knerr et al. showed that, upon DHT stimulation, DAAM2 forms nuclear actin-dependent transcriptional condensates together with the AR, and that the p.(Asn614ThrfsTer2) and p.(Asp165Glu) variants substantially impair AR-mediated transcription [11]. Because the inguinoscrotal phase of testicular descent strictly requires functional AR signaling [2,6], attenuation of DAAM2-mediated AR coactivation could plausibly result in incomplete testicular descent. This mechanistic link is consistent with the clinical phenotype of partial androgen insensitivity syndrome, in which cryptorchidism and hypospadias frequently coexist as manifestations of disrupted fetal androgen action [1,11].

### 4.2. Comparison with established cryptorchidism-associated genes

Most nonsyndromic cryptorchidism is not explained by monogenic variants in the principal descent-pathway genes. Mutations in *INSL3* and its receptor RXFP2, which drive the transabdominal phase, are found in only a small percentage of patients, and reported associations have been inconsistent across populations [2,6,7]. Coding AR mutations are likewise uncommon in isolated cryptorchidism, even though AR signaling is indispensable for the inguinoscrotal phase [2,7]. Against this background, candidate genes that modulate AR activity, such as *DAAM2*, proposed here, are mechanistically attractive, but the field’s experience with INSL3, RXFP2, and AR shows that initial small-cohort signals frequently fail to replicate. *DAAM2* must be established through replication rather than asserted from a pilot sample.

### 4.3. Evaluation of the p.(Asn614ThrfsTer2) variant

In our cohort, heterozygous p.(Asn614ThrfsTer2) carriage was observed approximately five times more frequently in cryptorchidism patients (12.5%) than in healthy controls (2.6%). Univariate logistic regression yielded an OR of 5.43; however, the CI was wide and included the null value (0.60–48.78), preventing the attainment of statistical significance (p = 0.131). Rather than providing evidence of no association, this pattern is most consistent with a type II error secondary to limited statistical power, a well-recognized limitation of rare-variant genetic association studies with modest sample sizes. Given the low expected heterozygote frequency in the control group (below 3%) and the small size of our cohort (n = 79), detection of an OR of 5 at a two-sided α-value of 0.05 would require substantially larger samples.

The p.(Asn614ThrfsTer2) frameshift is predicted to generate a truncated DAAM2 protein lacking the entire FH2 domain, which is essential for actin nucleation and processive elongation [9,11]. Loss of the FH2 domain is therefore expected to abolish the canonical formin activity of DAAM2 and, consequently, its capacity to assemble AR-associated transcriptional condensates at the nuclear level [11]. From a mechanistic perspective, a fivefold enrichment of a putative loss-of-function allele in a phenotype tightly linked to AR signaling is biologically plausible and merits further evaluation in larger independent cohorts.

### 4.3. Evaluation of the p.(Asp165Glu) variant

The p.(Asp165Glu) missense variant, located within the GTPase-binding domain of *DAAM2*, displayed a distinct pattern. Heterozygous carrier frequencies were effectively identical in the two groups (7.5% vs. 7.7%; OR: 1.03), indicating that heterozygous p.(Asp165Glu) did not confer a detectable risk for cryptorchidism in our population. However, the homozygous genotype was identified in 2 of 40 cryptorchidism patients (5.0%) and in none of the 39 controls. The absence of homozygous carriers in the reference group resulted in complete separation in the logistic regression model, precluding reliable estimation of an OR.

Although this observation does not, on its own, constitute proof of a statistical association, it suggests that the p.(Asp165Glu) variant may play a role in the etiology of the disease. Formins function as obligate homodimers, and homozygous expression of a functionally compromised allele may impact AR-dependent transcription more severely than the heterozygous state, in which a wild-type allele can partially compensate. Knerr et al. reported that the p.(Asp165Glu) variant retained the ability to colocalize with the AR but showed significantly reduced DHT-induced transcriptional activity [11]. A recessive or dose-dependent mode of inheritance is therefore conceivable, analogous to the recessive inheritance documented for biallelic *DAAM2* variants in steroid-resistant nephrotic syndrome type 24 [9]. Segregation analysis of the parents of the two homozygous patients is an essential next step to clarify this hypothesis.

### 4.4. *DAAM2* and broader male reproductive health

Cryptorchidism and hypospadias are considered complementary manifestations of the proposed testicular dysgenesis syndrome, which also encompasses poor semen quality and testicular germ cell tumor in adulthood and is hypothesized to share a common origin in disrupted fetal androgen action [1]. The identification of DAAM2 as an AR coregulator, together with reports of *DAAM2* structural variation in 46,XY disorders of sex development and functional *DAAM2* variants in partial androgen insensitivity with hypospadias, positions DAAM2 within the androgen signaling axis relevant to cryptorchidism [10,11]. Our findings suggest that variation in the *DAAM2* gene may also contribute to the isolated cryptorchidism phenotype.

In terms of population prevalence, both variants are rare in reference databases: in gnomAD v4, the global minor allele frequency of the p.(Asp165Glu) variant is approximately 0.002% and no homozygous cases have been reported, while the p.(Asn614ThrfsTer2) variant is not included in the database. The higher frequencies observed in our regional cohort (minor allele frequency of p.(Asp165Glu) in the control group: 3.85%; carrier frequency of p.(Asn614ThrfsTer2): 2.6%) may reflect the significant underrepresentation of the Eastern Black Sea population in global databases and possible regional or founder enrichment. These regional frequency estimates should ideally be validated in larger phylogenetically matched cohorts using orthogonal sequencing.

### 4.5. Strengths and limitations

The present study has several strengths. It is, to our knowledge, the first investigation of *DAAM2* variants in a cryptorchidism cohort. The study population was geographically homogeneous (from a single province), and cases and controls were of the same ethnicity and matched for sex and age range. We focused on two variants with established functional consequences on AR signaling [11], which provides mechanistic relevance to any observed genotype–phenotype relationship. The use of two complementary validated genotyping methods (TaqMan allelic discrimination and ARMS-PCR with high-resolution melting curve analysis) provided robust genotype calls.

However, several limitations must be acknowledged. First, the sample size (n = 79) was modest, constituting the principal limitation of the study. Low genotype frequencies required the use of exact statistical methods and resulted in wide CIs in the regression model. Second, the control group was drawn from children referred for *MEFV* gene testing, which may have introduced selection bias, although all members of the control group were screened for the absence of personal and family histories of cryptorchidism, hypospadias, or related urogenital anomalies. Third, parental samples for segregation analysis were not available at the time of this analysis; obtaining these samples, particularly for the two p.(Asp165Glu) homozygous probands, will be a priority in subsequent work. Fourth, additional coding and regulatory *DAAM2* variants beyond the two functionally characterized variants evaluated here were not screened; comprehensive sequencing of the *DAAM2* locus in a larger cohort would provide a more complete picture of *DAAM2* variation in cryptorchidism. Fifth, although baseline clinical characteristics have been provided (Table 1), the small number of variant carriers precludes a formal genotype–phenotype correlation. Sixth, all participants resided in Ordu Province in Türkiye’s Eastern Black Sea region and were of Turkish origin. Since the control group was selected from a clinically referred population rather than a general population sample, there is a possibility of selection bias.

### 4.6 . Future directions

Our findings should be interpreted as hypothesis-generating and require replication in independent, larger, and preferably multicenter cohorts. Functional assays examining the impact of the p.(Asp165Glu) homozygous state on AR transcriptional activity and on cells of the gubernacular lineage would strengthen the biological rationale of the present study. Comprehensive *DAAM2* sequencing, including intronic and regulatory regions, may identify additional variants contributing to cryptorchidism risk. Finally, integration of *DAAM2* genotype data with hormonal profiles (e.g., INSL3, testosterone, anti-Müllerian hormone) would help to delineate genotype–phenotype correlations within this clinically heterogeneous condition. In the long term, clarifying the role of *DAAM2*-related genetic susceptibility in larger prospective cohorts might provide pediatric surgeons with additional insight for risk stratification or individualized follow-up strategies in cryptorchidism.

## 5. Conclusion

In this pilot case-control study, no statistically significant association was demonstrated between the *DAAM2* variants p.(Asp165Glu) and p.(Asn614ThrfsTer2) and pediatric cryptorchidism. The numerically higher frequency of p.(Asn614ThrfsTer2) heterozygous carriage and the exclusive occurrence of p.(Asp165Glu) homozygosity in patients are preliminary hypothesis-generating observations whose nonsignificance is consistent with the limited statistical power of a pilot sample. Larger and adequately powered multicenter studies with appropriately sampled population controls are needed to determine whether *DAAM2* variation contributes to cryptorchidism.

## Figures and Tables

**Table 1:** Post hoc power analysis and required sample size for each variant.

Variant	Patient variant genotype n/N	Control variant genotype n/N	Cohen’s h	Current power	Probability of a Type II error	Number of groups required for 80% power	Number of groups required for 90% power
pN614pdfx2	5/40	1/39	0.401	0.430	0.570	98	131
pD165E	5/40	3/39	0.161	0.110	0.890	609	815

Power analysis was performed using a two-proportion comparison method based on the variant genotype model. The presence of a variant genotype was defined as the presence of a heterozygous or homozygous genotype. The required sample sizes are provided for each group. The probability of a type II error was calculated as 1 – power.

**Table 2:** Baseline clinical characteristics of the cryptorchidism cohort (n = 40) by *DAAM2* genotype.

Characteristic	All cases (n = 40)	p.(Asp165Glu) Carriers (n = 5)	p.(Asp165Glu) Wild-type (n = 35)	p.(Asn614ThrfsTer2) Carriers (n = 5)	p.(Asn614ThrfsTer2) Wild-type (n = 35)
**Age at diagnosis, months**, Mean ± SD	25.7 ± 24.6	-	-	-	-
**Age at surgery, months**, Mean ± SD	26.78±24.58	-	-	-	-
Unilateral, n (%)	30 (75.0%)	4 (80%)	26 (74.3%)	3 (60%)	27 (77.1%)
Bilateral, n (%)	10 (25.0%)	1 (20%)	9 (25.7%)	2 (40%)	8 (22.9%)
Right, n/N unilateral (%)	22/30 (73.3%)	4/4 (100%)	18/26 (69.2%)	2/3 (66.7%)	20/27 (74.1%)
Left, n/N unilateral (%)	8/30 (26.7%)	0/4 (0%)	8/26 (30.8%)	1/3 (33.3%)	7/27 (25.9%)
**Testicular position**					
Inguinal, n (%)	39 (97.5%)	4 (80%)	35 (100%)	5 (100%)	34 (97.1%)
Intraabdominal (nonpalpable), n (%)	1 (2.5%)*	1 (20%)*	0 (0%)	0 (0%)	1 (2.9%)

SD: Standard deviation.

**Table 3:** Hardy–Weinberg equilibrium analysis of genetic variants in the control group.

Variant	Genotype	Observed n	Expected n	Exact HWE p
pD165E	Wild/homozygous reference	36	36.06	1.000
pD165E	Heterozygous	3	2.88	1.000
pD165E	Homozygous variant	0	0.06	1.000
pN614pdfx2	Wild/homozygous reference	38	38.01	1.000
pN614pdfx2	Heterozygous	1	0.99	1.000
pN614pdfx2	Homozygous variant	0	0.01	1.000

HWE: Hardy–Weinberg equilibrium. HWE analysis was performed only for the control group. Expected genotype counts were calculated using the allele frequencies in the control group. Due to low cell frequencies, HWE was assessed using the exact test.

**Table 4:** Genotype distributions and linear-by-linear association analysis of *DAAM2* variants in the cryptorchidism group and healthy control group.

Genotype	Healthy control (n = 39)	Cryptorchidism (n = 40)	*χ* *^2^* *LBL*	p-value
***p.(Asn614ThrfsTer2)***			2.743	0.201^a^
Wild-type	38 (97.4%)	35 (87.5%)		
Heterozygous	1 (2.6%)	5 (12.5%)		
***p.D165E***			1.163	0.423^a^
Wild-type	36 (92.3%)	35 (87.5%)		
Heterozygous	3 (7.7%)	3 (7.5%)		
Homozygous	0 (0.0%)	2 (5.0%)		

Percentages are presented as column percentages.

χ^2^ LBL denotes the linear-by-linear association test statistic.

a Due to data sparsity in cross-tabulations (low expected frequencies), exact p-values are reported instead of asymptotic values.

**Table 5:** Binary logistic regression estimates of the association between *DAAM2* genotypes and risk of cryptorchidism.

Genotype	OR (95% CI)	p-value
***p.(Asn614ThrfsTer2)***		
Wild-type	Reference	—
Heterozygous	5.43 (0.60–48.78)	0.131
***p.D165E***		
Wild-type	Reference	—
Heterozygous	1.03 (0.19–5.45)	0.974
Homozygous	NC^b^	0.999

OR: Unadjusted odds ratio; CI: confidence interval; NC: not calculable.

b Due to the absence of homozygous carriers in the healthy control group, complete separation occurred in the logistic regression model; a reliable risk coefficient could therefore not be estimated for this subgroup.

**Table 6:** Evaluation of the effect of genetic variants on the risk of undescended testis using exact and Firth logistic regression methods.

Genotype	Control (n)	Cryptorchidism (n)	Fisher exact OR (%95 CI)	Fisher exact p	Exact logistics OR (95% CI)	Exact logistics p	Firth OR (%95 CI)	Firth p
**pN614pdfx2**								
Wild	38	35	Reference		Reference		Reference	
Heterozygous	1	5	5.33 (0.56–63.02)	0.201	4.53 (0.50–228.78)	0.225	3.97 (0.74–40.17)	0.111
Homozygous	0	0	NC*	-	NC*	-	NC*	-
**pD165E**								
Wild	36	35	Reference		Reference		Reference	
Heterozygous	3	3	1.03 (0.13–8.21)	1.000	1.08 (0.12–10.25)	1.000		0.972
Homozygous	0	2	∞ (0.18–∞)	0.493	2.31 (0.17–∞)	0.479	5,12 (0.40–719.56)	0.228

Fisher exact p-value for the overall genotype distribution of pD165E: 0.595.

OR: Odds ratio; CI: confidence interval; NC: not calculated.

* pN614pdfx2 OR could not be calculated because no homozygous carrier was detected.

In the pD165E homozygous comparison, since there were no homozygous individuals in the control group, the Fisher exact OR was calculated as infinite. The upper confidence limit in exact logistic regression is infinite. Firth penalized logistic regression produced a finite effect estimate; however, due to the sparse cell structure, wide confidence intervals, and convergence problems for previous Firth outputs, the results should be interpreted at the hypothesis-generating level.
